# Protistan Diversity in the Arctic: A Case of Paleoclimate Shaping Modern Biodiversity?

**DOI:** 10.1371/journal.pone.0000728

**Published:** 2007-08-15

**Authors:** Thorsten Stoeck, Jennifer Kasper, John Bunge, Chesley Leslin, Valya Ilyin, Slava Epstein

**Affiliations:** 1 Department of Ecology, University of Kaiserslautern, Kaiserslautern, Germany; 2 Department of Statistical Science, Cornell University, Ithaca, New York, United States of America; 3 Department of Biology, Northeastern University, Boston, Massachusetts, United States of America; 4 Marine Science Center, Northeastern University, Nahant, Massachusetts, United States of America; University of Ottawa, Canada

## Abstract

**Background:**

The impact of climate on biodiversity is indisputable. Climate changes over geological time must have significantly influenced the evolution of biodiversity, ultimately leading to its present pattern. Here we consider the paleoclimate data record, inferring that present-day hot and cold environments should contain, respectively, the largest and the smallest diversity of ancestral lineages of microbial eukaryotes.

**Methodology/Principal Findings:**

We investigate this hypothesis by analyzing an original dataset of 18S rRNA gene sequences from Western Greenland in the Arctic, and data from the existing literature on 18S rRNA gene diversity in hydrothermal vent, temperate sediments, and anoxic water column communities. Unexpectedly, the community from the cold environment emerged as one of the richest observed to date in protistan species, and most diverse in ancestral lineages.

**Conclusions/Significance:**

This pattern is consistent with natural selection sweeps on aerobic non-psychrophilic microbial eukaryotes repeatedly caused by low temperatures and global anoxia of snowball Earth conditions. It implies that cold refuges persisted through the periods of greenhouse conditions, which agrees with some, although not all, current views on the extent of the past global cooling and warming events. We therefore identify cold environments as promising targets for microbial discovery.

## Introduction

Periods of steady climate on our planet have been punctuated by extraordinary paleoclimate events, from extreme greenhouses, with Arctic ocean temperatures soaring above 20°C [1], to the freezing conditions of snowball Earth [2]–[4]. These dramatic fluctuations in temperature, and global anoxia that likely coincided with the freezing events [5], must have had a pronounced effect on the evolution of biological diversity. Each event probably acted as a powerful natural selection filter, with different events selecting for exactly opposite traits. It is possible that linking the history of paleoclimate to evolution of biodiversity may help to explain the present pattern of this biodiversity. We are interested in what biodiversity predictions can be made based on the paleoclimate dynamics, and whether such predictions can be verified. Here we examine a link between the diversity of microbial eukaryotes and paleoclimate events over the course of their evolution.

There is increasing evidence that several snowball Earth conditions occurred in the history of our planet (e.g., in the Paleoproterozoic 2.4 billion years ago [6], and more recently in the Neoproterozoic, 710 and 635 million years ago [2]–[3], [5], [7]), as did the ultra-greenhouse temperatures that likely followed such conditions [2], [5], and more moderate greenhouse events, the latest of which occurred in the Cenozoic 55 million years ago [1], [8]. Regardless of when and at what temperature the eukaryotes originated, they must have survived dramatic temperature and oxygen level fluctuations. Extreme cold would obviously select for extreme psychrophiles, making all other organisms less competitive. The opposite would be expected during extreme global warming periods. We postulate that one principal factor determining what kind of biodiversity would survive a cataclysmic shift is the presence or absence of a refuge. Depending on whether or not temperature refuges persisted throughout the history of eukaryotic life, we can envision four scenarios of how paleoclimate might-and in fact should–have influenced the evolution of microbial eukaryotes:

1. Refuges did not exist, and temperature extremes reached every corner of the biosphere. If so, neither psychrophiles nor thermophiles would be expected to survive an adverse temperature swing, and would have to evolve anew after each such event.

2. Only cold refuges persisted through time. If so, today's cold environments would be enriched with basal lineages with a long history of continuous evolution in the cold. Thermophilic lineages of today would be descendants of psychrophiles. Having evolved after the last global freezing event, these lineages would be expected to root within clades of psychrophilic origin.

3. Only hot refuges persisted through time. This would lead to a scenario opposite to #2: assemblages in hot environments would be enriched with basal lineages, with psychrophiles appearing as evolutionary newcomers.

4. Both types of refuges continuously existed throughout the history of eukaryotic life. In this case, today both low- and high-temperature environments would be expected to show a diversity of microeukaryotic life, each characterized by its own basal lineages. Continuous evolution of cold adaptations in thermophiles would produce evolutionarily young psychrophilic lineages within clades of thermophilic origin, and vice versa, leading to clades represented by mixes of thermo- and psychrophiles.

Two additional considerations add to the picture of predicted patterns of microeukaryotic diversity. First, since the snowball Earth was largely anoxic, the majority of survivors must have been obligate and facultative anaerobes. One would expect this to manifest today as a diversity of deeply rooted microbial eukaryotes with anaerobic life style. Second, it is reasonable to expect that older habitats would harbor larger biological richness by providing longer periods of time for this richness to evolve, especially since the diversification rate of microorganisms may have exceeded their extinction rate for billions of years [9]. This richness should be distinct from that of a quick explosive origin (*e.g.*, cichlid fish in Africa [10]): the first should appear as a collection of lineages rooted deeply, whereas the second as a number of short lineages branching off from a distinct node of relatively recent origin.

The four scenarios above are not all equally plausible. The first is the least realistic, because hydrothermal environments have likely been a persistent feature of the ocean floor since the origin of our planet [11]. Though rather ephemeral at individual sites, the hydrothermal vent environment in general would still provide a safe harbor for at least a selection of thermophilic (and perhaps some mesophilic) organisms, even at the peaks of the snowball Earth events.

In contrast, there is little evidence to support the persistence of cold refuges. The paleoceanographic record constructed from a sediment core from a recent drilling expedition in the Arctic Ocean suggests that early Cenozoic was very warm, with polar waters staying above 20°C year round, and no cold deep ocean [1], [12]. The mid-Cretaceous is also thought to have been exceptionally warm [13]–[14]. It appears that cold environments might have been completely–and repeatedly-wiped out during such times, making scenarios 2 and 4 rather unlikely.

This leaves case 3 as the most plausible, at least in terms of current paleoclimate views. This scenario implies that microbial eukaryotes at elevated temperatures, such as in hydrothermal vent environments, should be uniquely rich in ancestral lineages, and exhibit a relatively large number of species, especially with anaerobic lifestyle. In contrast, protistan assemblages in cold environments would be expected to have lower species diversity and to be represented by lineages of recent evolutionary origin. The peak of the latest extreme greenhouse event was approximately 55 million years ago [1], and glaciation in the northern hemisphere did not start until 6–10 million years ago [15]. Following the logic of the “center of origin” hypothesis [16], it seems reasonable to expect that protists from the polar regions, having evolved over that relatively short period of time, would form few (perhaps no) “old” unique clades, and would fall into groups characterized by deeply rooted thermophiles. Here we test these predictions by comparing the 18S rRNA gene diversity in two hydrothermal vent communities (HV1 [17] and HV2 [18]), to the diversity in an original dataset we obtained from a tidal flat in the Arctic (DI; Disko Island, Western Greenland). This sediment habitat represents one of the coldest tidal flats studied because it stays ice-free for only 3–4 months a year, with water temperature averaging −1.8°C [19], and rarely exceeding 4–6°C even on a sunny summer day. For comparative purposes, we include in our analyses the only available 18S rRNA surveys of temperate sediments (WH [20], and CA1 and CA2 [21]), as well as one of the best-studied (to date) protistan assemblages from an anoxic system (CB; Cariaco Basin in the Caribbean [22], [23], [24]). We compare the results of these surveys by examining (1) their respective overall species richness; (2) the presence/absence, phylogeny, and richness of unique clades of high taxonomic order; and (3) the biology of relevant organisms.

## Results and Discussion

### Species richness

The rRNA surveys in question were of unequal size, and the observed numbers of individual phylotypes detected were not directly comparable. We recently developed a statistical procedure to estimate biological richness based on observed data of this type, and tested it on both prokaryotic and protistan datasets [22], [25]. We applied this approach here to estimate the total richness of the samples from the target communities, which allows a direct comparison between such samples (Table 1). We note that the true richness of hydrothermal vent communities may be overestimated here because some of the 18S sequences detected at the richer site (HV1) likely originated from the photic zone of the overlying water column [18]. When the uppermost layer of the sediment was excluded from analyses, the number of allochthonous sequences was reduced [18]. We note that even though this lowered the estimate of total protistan richness (compare HV1 and HV2 in Table 1), it remained higher than those for temperate tidal flat. This may appear surprising because the ultimate community food base (i.e., fixed organic material) is likely less diverse and abundant in deep sea than in shallow water environments. However, the apparently higher richness of hydrothermal vent protistan communities can be easily explained if this environment was evolutionarily old, and its inhabitants had a long uninterrupted history of diversification.

**Table 1:** 18S rDNA phylotype richness estimates of protistan assemblages in Disko Island (DI, Arctic); hydrothermal vents HV1 [17] and HV2 [18]; temperate sediment communities WH [20], CA1 and CA2 [21]; and anoxic water column CB [23].

		DI (707)*	HV1 (271)*	HV2 (183)*	WH (66)*	CA1 (641)*	CA2 (700)*	CB (725)*
**OTUs at >99% gene sequence similarity**	Observed	238	90	n/a	36	n/a	n/a	107
	Total richness, parametric estimate±SE	605±73	578±173		48±6			398±156
	Abundance model	2-mixed exponential	2-mixed exponential		**			2-mixed exponential
	Total richness, nonparametric estimate±SE	570±80	674±194					311±84
	Estimator	ACE1	ACE1					ACE1
**OTUs at >98% gene sequence similarity**	Observed	173	95	n/a	n/a	25	17	99
	Total richness, parametric estimate±SE	430±87	404±164			58±21	19±2	263±74
	Abundance model	Inverse Gaussian	2-mixed exponential			Pareto	2-mixed exponential	2-mixed exponential
	Total richness, nonparametric estimate±SE	345±50	358±100			77±40	17±0	228±54
	Estimator	ACE1	ACE1			ACE1	ACE1	ACE1
**OTUs at >97% gene sequence similarity**	Observed	149	121	33	n/a	n/a	n/a	91
	Total richness, parametric estimate±SE	308±37	329±125	71±18				230±65
	Abundance model	2-mixed exponential	2-mixed exponential	2-mixed exponential				2-mixed exponential
	Total richness, nonparametric estimate±SE	294±46	287±77	75±30				186±43
	Estimator	ACE1	ACE1					ACE1

n/a–data not available;

* library size (number of clones)

** data insufficient for richness estimation, numbers shown are lower bounds based on equal-OTU-sizes (unmixed Poisson) model.

Unexpectedly, the Arctic protistan community emerged as one of the richest in species to date. This contrasts with the prevailing general view that Arctic cold conditions do not favor high biological diversity [26], but is tentatively supported by recent findings of a latitudinal increase in eukaryotic soil richness towards the southernmost sampling points in Antarctica [27]. Considering the small size of our sediment sample for DNA extraction (3 g), and the fact that any estimate based on the rRNA approach is conservative due to the well-known PCR and primer biases [28], the 605 phylotypes predicted to co-exist in the sample tell a story of notable diversity and richness. A recently detected high diversity of planktonic protists in the Arctic [29] lends additional support to our findings.

While comparing the richness estimates (Table 1), we considered several factors that could bias these estimates, thus potentially limiting the usefulness of such comparison.

1. Different sites were sampled using somewhat different methodologies. Similarly to our research, one of the two temperate tidal flat studies used both anoxic and suboxic parts of the sediment cores [20], whereas the second used only the anoxic sediment zone [21]. Nonetheless, the richness estimates are essentially the same between the latter two (Table 1), and equally low relative to the one obtained here. This indicates that the presence of aerobic protists in our samples cannot explain the large difference between their richness and that in the temperate samples. This is in line with the growing understanding that protistan diversity in anoxic environments significantly exceeds that of oxic habitats [30]–[31].

2. Sand binds dissolved DNA [32], and the rRNA gene diversity uncovered here might contain signatures of allochthonous species. While there is a good chance this might be the case, the artifact in question is probably characteristic of all sediment works. The temperate and vent sediments considered in our analyses were all micro- to mesoporal sands, and all could be enriched with DNA from nearby sources. While assessing the impact of this artifact, it is important to consider the richness of the contributing sources. Temperate sediments must be influenced by, among other factors, adjacent soils, which are known to be rich in microbial life. In contrast, the contribution of this source in the present study was minimal, as the soils immediately adjacent to the sampling site were barren stony desert, with partial snow cover. The likely higher diversity of allochthonous DNA in temperate sediments makes the observed difference between protistan diversity in temperate vs. Arctic sediments conservative.

3. The original dataset was obtained using a multiple primer approach, which could detect more protistan rRNA genes than a more traditional single primer approach. This is an important difference between this and some (but not all) published research, and therefore deserves careful analysis. In addition to predicting the DI protistan richness based on pooled clone data (Table 1), we conducted separate analyses using rRNA gene sequences obtained with individual PCR primers. Using primer sets D1, 2, and 3 (Table 2), we obtained and sequenced 192 clones for each clone library, of which 143, 170, and 138, respectively, produced protistan 18S rRNA gene sequences. We grouped these into OTUs at 99% and 98% rRNA gene sequence identity, and obtained respectively 79 and 62 OTUs for clone D2 and D1 clone libraries, and 52 and 53 OTUs for D1 and D2 clone libraries. We then reconstructed the OTU frequency distribution in the above 4 groups of OTUs, and applied to these frequency distributions the same statistical approach we used with the pooled data (Table 1) to produce estimates of total protistan richness. Because these analyses use the data obtained with single primer sets, their results are comparable with those from the majority of published reports.

**Table 2:** Primer sequences used in this study for specific PCR-amplification of 18S rRNA gene sequences from environmental Disko Island DNA.

Primer set	Primer name		Sequence (5′-3′)	Reference
**D1** (nested reaction)	EukA (21F)	First reaction	AACCTGGTTGATCCTGCCAGT	[61]
	EukB (1791R)		TGATCCTTCTGCAGGTTCACCTAC	[61]
	Euk82F	Second reaction	GAA(AGT)°CTG(CT)°GAA(CT)*GGCTC	[62]
	U1391R		GGG CGG TGT GTA CAA (AG)*G(AG)*	[63]
**D2** (nested reaction)	EukA (21F)	First reaction	AACCTGGTTGATCCTGCCAGT	[61]
	EukB (1791R)		TGATCCTTCTGCAGGTTCACCTAC	[61]
	Euk360F	Second reaction	CGGAGA(AG)*GG(AC)*GC(AC)*TGAGA	[61]
	U1517R		ACG GCT ACC TTG TTA CGA CTT	[64]
**D3**	18S-6-Cil5-F		AA(CT)*CTGGTTGATCCTGCCAG	[65]
	18S-1101-Cil-R		GAT CC(AT)*TCTGCAGGTTCACCTAC	[65]
**D4**	Euk1438R		GGGCATCACAGACCTGTTAT	[66]
	Euk528F		CGGTAATTCCAGCTCC	[67]

* mixed base sites are designated according to IUB code. F = forward, R = reverse, Cil = ciliate specific, Euk = eukaryote specific, U = universal, numbers refer to *E. coli* 16S rRNA gene positions. The primer EukA is a forward primer referring to *S. cerevisiae* 18S rRNA gene position 2-22. The primer EukB is a reverse primer referring to *S. cerevisiae* 18S rRNA gene position 1795 to 1772.

As expected, predictions of protistan richness based on single PCR primer datasets were lower than those based on pooled clone data (Table 1). These predictions are: 225±72 (SE) OTUs at 99% level of gene identity based on primer set 3 individual dataset, and 126±42 (SE) and 132±30 (SE) OTUs at 98% level based on primer sets 1 and 2 individual datasets, respectively (the 4^th^ analysis that used primer set 2 at 99% level OTUs did not produce statistically valid total richness predictions). Though these estimates are substantially lower than those based on the pooled data (Table 1), it is important to note that they are higher than an estimate of protistan richness in a temperate tidal flat community [20], and twice as high as either of the two anoxic tidal flats we considered [21], even though the latter study employed a multiple PCR primer approach. The above estimates appear on a par with HV2 and, with sediment communities in mind, are only second to HV1 (Table 1). It should be noted, however, that the HV1 data [17] contains a significant number of signatures of photosynthetic protists and is believed to be artificially enriched with allochthonous species from the overlying water column [18]. Therefore, the data obtained here, whether considered as a library based on the multiple PCR primer approach, or as a collection of libraries each based on an individual PCR primer, indicate a surprising level of protistan richness in the Arctic community.

We conclude that the evidence for this richness is not due to methodological differences between this and prior studies of 18S rRNA gene diversity in sediment communities, but rather points to the Polar environment as a “hot spot” of protistan biodiversity. This does not support the idea that this community is evolutionarily young and principally derived from thermophilic ancestors.

### Comparative phylogeny of Arctic microbial eukaryotes

**Figure 1:**
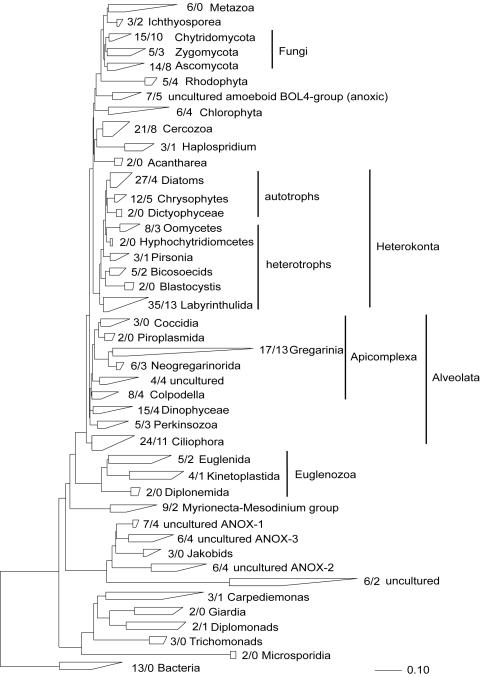
18S rDNA Maximum Parsimony (MP) tree showing the assignment of Disko Island phylotypes to major eukaryote clades. The numbers a/b indicate the total number of GenBank sequences representing the clade/the number of phylotypes detected. Ancestral sequences to a specific clade were included in the clade itself. Only protistan and fungal sequences are shown in the tree. Detailed phylogenies (partial treeing analyses) can be found in Figures 2–10.

**Figure 2:**
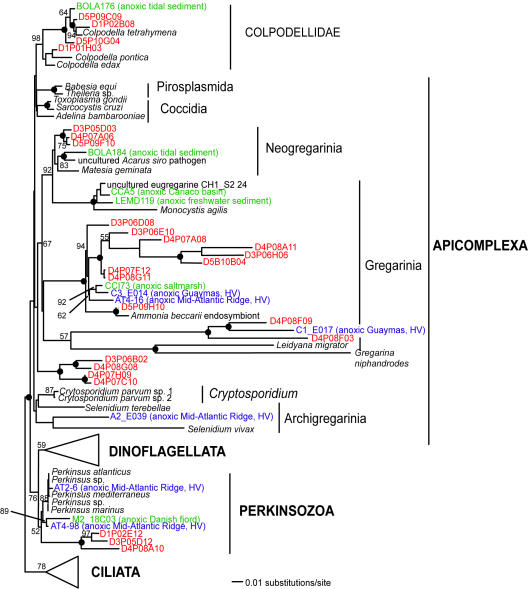
Minimum evolution phylogenetic tree of 18S rDNA sequences showing the position of apicomplexan and perkinsozoan Disko Island phylotypes (Alveolata). The tree was constructed under Maximum Likelihood criteria (ML) using a General Time Reversible (GTR) DNA substitution model with the variable-site gamma distribution shape parameter (G) at 0.6830 and the proportion of invariable sites (I) at 0.1125, and is based on 784 unambiguously aligned and conserved positions. Distance bootstrap values over 50% from an analysis of 1000 bootstrap replicates are given at the respective nodes; dots identify nodes with 100% bootstrap support. Clone names in red, blue, and green identify sequences reported in this study (DI) and from hydrothermal vent and temperate environments, respectively. The first two identifiers of the DI sequences (D1–D5) designate the different PCR primer sets used in this study (detailed information in Table 2).

The phylogenetic diversity of microbial eukaryotes from a marine tidal flat in Western Greenland is substantial, matching or exceeding that detected in hydrothermal vents [17]–[18], temperate sediments [20]–[21], or any other community studied to date [33]–[35]. We uncovered representatives of all major protistan clades known to science, except microsporidians, trichomonads, and uncultured Alveolates (Figure 1; for a detailed account of the phylogenetic positions of all 18S rRNA gene sequences discovered see Figures 2–10).

**Figure 3 pone-0000728-g003:**
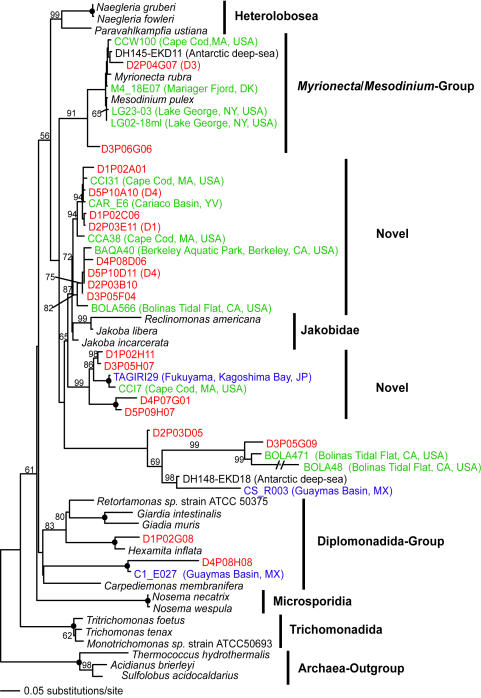
Minimum evolution phylogenetic tree of 18S rDNA sequences showing the position of basal-branching Disko Island sequences. The tree was constructed under Maximum Likelihood criteria (ML) using a General Time Reversible (GTR) DNA substitution model with the variable-site gamma distribution shape parameter (G) at 1.3667, the proportion of invariable sites (I) at 0.0658, and base frequencies and a rate matrix for the substitution model as suggested by Modeltest (see Material and Methods), based on 553 unambiguously aligned and conserved positions. Because of their uncertain position in phylogenetic 18S rDNA sequence trees, the branching orders of the primitive jakobid flagellates and the *Myrionecta*/*Mesodinium* (Ciliophora) group are not supported. For a detailed explanation of the basal branching of the latter group see e.g. [31], [68]. Distance bootstrap values over 50% from an analysis of 1000 bootstrap replicates are given at the respective nodes; dots identify nodes with 100% bootstrap support. Clone names in red, blue, and green identify sequences reported in this study (DI) and from hydrothermal vent and temperate environments, respectively. The first two identifiers of the DI sequences (D1–D5) designate the different PCR primer sets used in this study (detailed information in Table 2).

**Figure 4 pone-0000728-g004:**
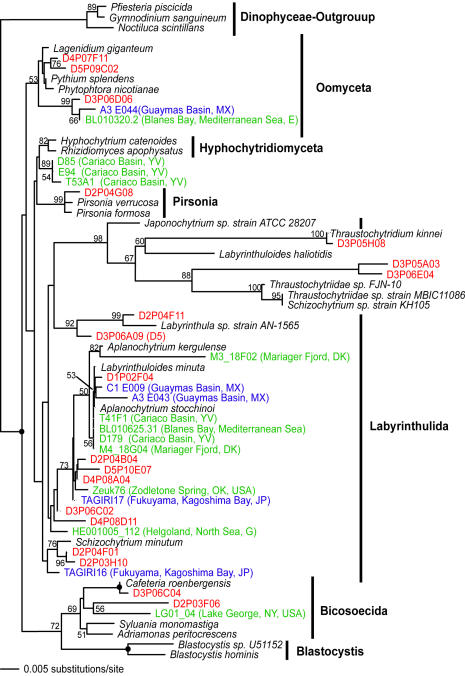
Minimum evolution phylogenetic tree of 18S rDNA sequences showing the position of heterotrophic stramenopile Disko Island sequences. The tree was constructed using a Tamura Nei DNA substitution model with the variable-site gamma distribution shape parameter (G) at 0.6830 and the proportion of invariable sites (I) at 0.1125, and is based on 784 unambiguously aligned and conserved positions. Distance bootstrap values over 50% from an analysis of 1000 bootstrap replicates are given at the respective nodes; dots identify nodes with 100% bootstrap support. Clone names in red, blue, and green identify sequences reported in this study (DI) and from hydrothermal vent and temperate environments, respectively. The first two identifiers of the DI sequences (D1–D5) designate the different PCR primer sets used in this study (detailed information in Table 2).

**Figure 5 pone-0000728-g005:**
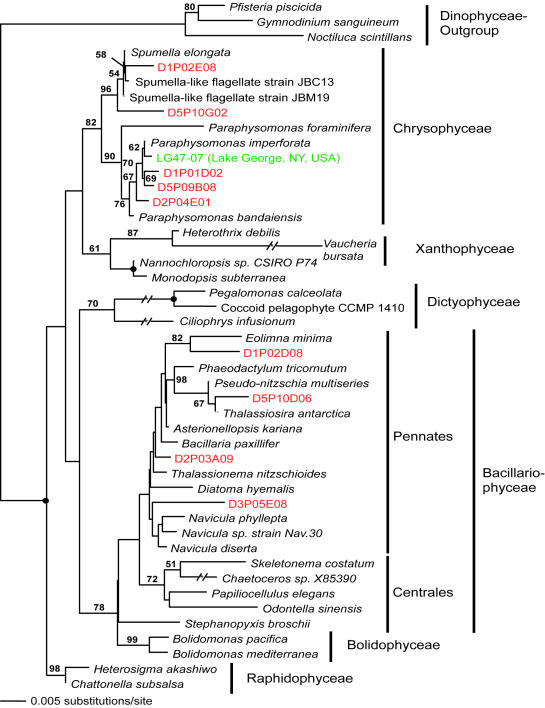
Minimum evolution phylogenetic tree of 18S rDNA sequences showing the position of autotrophic stramenopile Disko Island sequences. The tree was constructed under using a Tamura Nei substitution model with the variable-site gamma distribution shape parameter (G) at 0.7606 and the proportion of invariable sites (I) at 0.4458, and is based on 851 unambiguously aligned and conserved positions; dots identify nodes with 100% bootstrap support. Clone names in red, blue, and green identify sequences reported in this study (DI) and from hydrothermal vent and temperate environments, respectively. The first two identifiers of the DI sequences (D1–D5) designate the different PCR primer sets used in this study (detailed information in Table 2).

**Figure 6 pone-0000728-g006:**
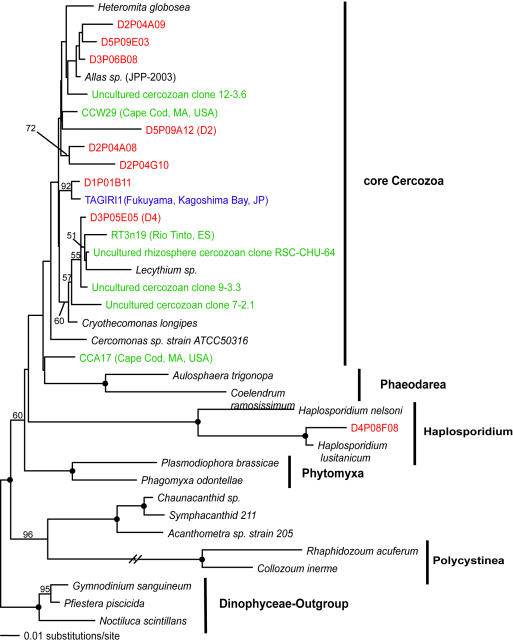
Minimum evolution phylogenetic tree of 18S rDNA sequences showing the position of rhizarian Disko Island sequences. The tree was constructed under Maximum Likelihood criteria (ML) using a General Time Reversible (GTR) DNA substitution model with the variable-site gamma distribution shape parameter (G) at 0.8312 and the proportion of invariable sites (I) at 0.2533, and is based on 817 unambiguously aligned and conserved positions. Distance bootstrap values over 50% from an analysis of 1000 bootstrap replicates are given at the respective nodes; dots identify nodes with 100% bootstrap support. Clone names in red, blue, and green identify sequences reported in this study (DI) and from hydrothermal vent and temperate environments, respectively. The first two identifiers of the DI sequences (D1–D5) designate the different PCR primer sets used in this study (detailed information in Table 2).

**Figure 7 pone-0000728-g007:**
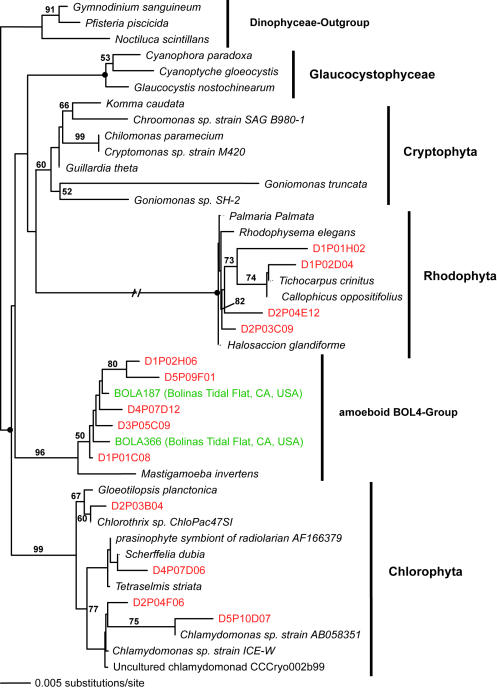
Minimum evolution phylogenetic tree of 18S rDNA sequences showing the position of unicellular algal Disko Island sequences (Plantae). The tree was constructed using a Tamura Nei DNA substitution model with the variable-site gamma distribution shape parameter (G) at 0.6123, and is based on 813 unambiguously aligned and conserved positions. Distance bootstrap values over 50% from an analysis of 1000 bootstrap replicates are given at the respective nodes; dots identify nodes with 100% bootstrap support. Clone names in red, blue, and green identify sequences reported in this study (DI) and from hydrothermal vent and temperate environments, respectively. The first two identifiers of the DI sequences (D1–D5) designate the different PCR primer sets used in this study (detailed information in Table 2).

**Figure 8 pone-0000728-g008:**
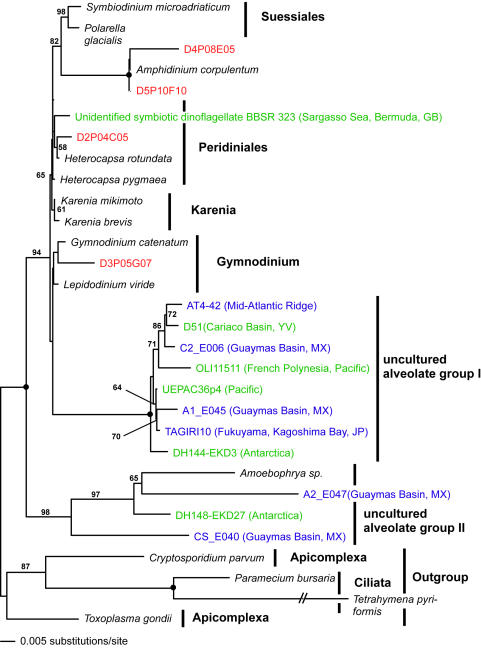
Minimum evolution phylogenetic tree of 18S rDNA sequences showing the position of dinophycean Disko Island sequences. The tree was constructed using a Tamura Nei DNA substitution model with the variable-site gamma distribution shape parameter (G) at 0.7606 and the proportion of invariable sites (I) at 0.4458, and is based on 851 unambiguously aligned and conserved positions. Distance bootstrap values over 50% from an analysis of 1000 bootstrap replicates are given at the respective nodes; dots identify nodes with 100% bootstrap support. Clone names in red, blue, and green identify sequences reported in this study (DI) and from hydrothermal vent and temperate environments, respectively. The first two identifiers of the DI sequences (D1–D5) designate the different PCR primer sets used in this study (detailed information in Table 2).

**Figure 9 pone-0000728-g009:**
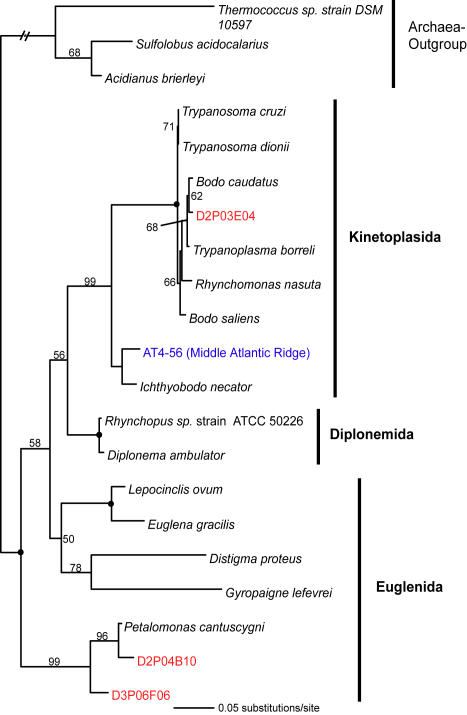
Minimum evolution phylogenetic tree of 18S rDNA sequences showing the position of euglenozoan Disko Island sequences. The tree was constructed under Maximum Likelihood criteria (ML) using a General Time Reversible (GTR) DNA substitution model with the variable-site gamma distribution shape parameter (G) at 0.8904 and the proportion of invariable sites (I) at 0.1517, and is based on 730 unambiguously aligned and conserved positions. Distance bootstrap values over 50% from an analysis of 1000 bootstrap replicates are given at the respective nodes; dots identify nodes with 100% bootstrap support. Clone names in red, blue, and green identify sequences reported in this study (DI) and from hydrothermal vent and temperate environments, respectively. The first two identifiers of the DI sequences (D1–D5) designate the different PCR primer sets used in this study (detailed information in Table 2).

**Figure 10:**
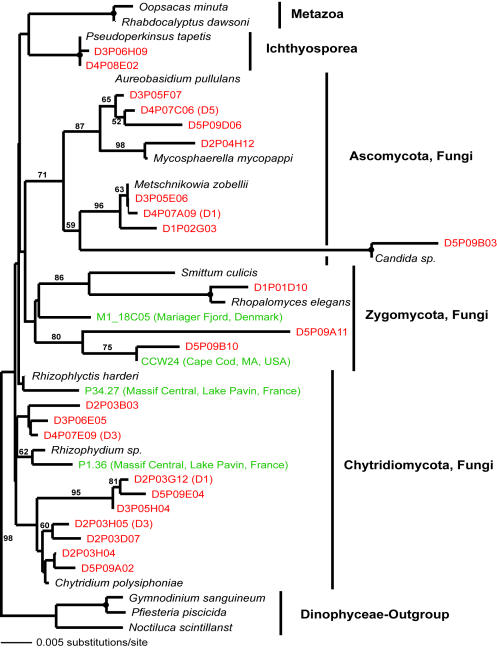
Minimum evolution phylogenetic tree of 18S rDNA sequences showing the position of ophistokont Disko Island sequences. The tree was constructed using a Tamura Nei DNA substitution model with the variable-site gamma distribution shape parameter (G) at 0.6200, and is based on 669 unambiguously aligned and conserved positions. Distance bootstrap values over 50% from an analysis of 1000 bootstrap replicates are given at the respective nodes; dots identify nodes with 100% bootstrap support. Clone names in red, blue, and green identify sequences reported in this study (DI) and from hydrothermal vent and temperate environments, respectively. The first two identifiers of the DI sequences (D1–D5) designate the different PCR primer sets used in this study (detailed information in Table 2).

Apart from its sheer breadth, one interesting aspect of the DI phylotypes' diversity is that it is skewed in favor of several specific taxonomic groups. For example, apicomplexans are represented remarkably well in our Arctic clone libraries and constituted over 12% of all unique protistan rRNA gene sequences. This proportion is the highest ever recorded; it exceeds the second highest by 5-fold (2.4%; temperate intertidal community [21]). Further statistical analyses estimated the total number of distinct apicomplexan OTUs (>99% sequence identity) at the Arctic site to be 174±60, exceeding the entire protistan richness at any of the three temperate tidal flats studied to date (Table 1). Perhaps even more importantly, a number of the Arctic apicomplexan lineages are deeply rooted within the clade (Figure 2), which makes the hypothesis of their recent diversification implausible.

Similarly, chytrid fungi, absent from the hydrothermal or temperate sediment sites studied so far, were well represented in the DI data and constituted almost 5% of all unique 18S rRNA gene sequences detected there. We estimated the total number of OTUs (>99% sequence identity) of all fungi to be 60±22; the data on chytrid fungi alone was insufficient for direct richness estimation, but a conservative lower bound (based on the equal-OTU-abundance or unmixed Poisson model) for the number of chytrid fungi OTUs was 14±2. Interestingly, the only other study of 18S rRNA gene diversity in a polar terrestrial environment (Antarctic soil, [27]) also noted an elevated diversity of chytrid fungi. Similarly to apicomplexans, chytrid lineages uncovered here do not seem to be a result of recent radiation: they either form novel sister clades to the known chytrids, or appear ancestral to them (Figure 9).

Jakobid-like organisms showed a strong presence in Arctic as well, constituting over 5% of all unique 18S rRNA sequences (Figure 3). However, they are notably absent at hydrothermal sites. Also noteworthy is a substantial diversity of Labyrinthulida in the DI data, some of which appear ancestral to the Labyrinthulids detected at hydrothermal vent sites (Figure 4).

We note that those clades that appear disproportionately rich in the Arctic are also evolutionarily older. Labyrinthulids are almost certainly ancestral to one of the largest protistan clades, Stramenopiles [36]. Chytrids are undoubtedly primitive and are thought to be ancestral to Fungi [37]. Apicomplexans possess a specialized apical complex suggesting their derived nature, but other ultrastructural characteristics point to their position as ancestral to another large eukaryotic clade, the Alveolates [38]–[39]. Most interestingly, Jakobids are characterized by the most bacteria-like mitochondrial genome known, and are regarded as the most ancestral mitochondria-bearing eukaryotes [40]–[42]. Collectively, Stramenopiles, Alveolates, Fungi, and Jakobids represent a substantial part of the entire eukaryotic diversity known today, and finding an extensive richness of their ancestral lineages in one community identifies this community as an important target for future efforts, and potentially informative for reconstructing eukaryotic evolution.

This comparative phylogenetic analysis leads to an inescapable conclusion: not only does the Arctic appear to be a “hot spot” of protistan biodiversity, but it is also notably rich in lineages ancestral to several principal eukaryotic clades, if not to the eukaryotes in general. It seems unlikely that communities with such traits are evolutionarily young and derived (*e.g.*, from thermophiles). A simpler explanation is that communities in the cold environment of the Arctic have been evolving in this or a similar environment for a substantial period of time, possibly comparable to the age of eukaryotes. This suggests that cold refuges might have persisted even through the warmest periods of the last one to two billion years, which is in conflict with the current view on the extent of global warming episodes ([1], but see also [43]).

### Biology of Arctic microbial eukaryotes

The four clades that are significantly–if not uniquely–rich in the Arctic sediment clone libraries are Apicomplexans, Chytrids, Labyrinthulids, and Jakobids. All these organisms are heterotrophs; many are saprophytes or parasites. Interpreting these observations meets with two difficulties.

First, the source of organic material necessary to support such a diversity of heterotrophs is not obvious. Autochthonous material is a doubtful source of organics given the uncharacteristically low number of autotrophic protists detected. Seaweeds are typically absent from Arctic tidal flats because of the action of winter ice. Diatoms and uncultured alveolates, which are ubiquitous components of the water column communities, mayrepresent a potential source of carbon, but this is hypothetical as our (sediment) dataset contains few to no signatures of these organisms. The source of allochthonous energy supply is also unclear. The sampling site is surrounded by a stony arctic desert with small patches of high latitude tundra, an unlikely supplier of organic material. By exclusion, this leads to subtidal fields of seaweeds, dominated principally by brown and red algae of substantial diversity (over 100 species in Greenland, [44]), as potential suppliers of carbon. We hypothesize that algal biomass which has washed ashore, together with drift seaweeds, constitute an important source of “missing” energy. This would explain why so many of the protists detected belong to groups rich in saprophytes (*e.g.,* Chytrids, Labyrinthulids).

The second difficulty is to explain an unparalleled richness of Apicomplexans, a group with very few known free-living representatives. We hypothesize that the apicomplexan life cycle, which includes resilient cysts, makes this group particularly competitive, and thus particularly successful, in the harsh environment of the Arctic. However, this cannot expect the puzzling aspect of high apicomplexan diversity because the Arctic has a low diversity of prospective hosts (animals, primarily mammals). Benthic macrofauna is also known to decrease in diversity in the northern direction [16], and it is reasonable to think the diversity of their parasites would mirror the trend. If so, the diversity of parasitic apicomplexans and chytrids would be expected to be higher in the temperate zone, not in Disko Bay. Our data show a strong opposite trend. The simplest explanation is that our rRNA survey detected a diversity of previously unknown free-living Apicomplexans and chytrids. Some of them may be close to the free-living ancestor of the present day parasitic forms, which is line with our finding deeply rooted apicomplexan and chytrid lineages. This is also indirectly supported by our detection of several 18S rRNA gene sequences related to *Colpodella*, a rare example of free-living apicomplexan, which is thought to be ancestral to the clade [45]–[46].

One important common theme in the biology of species from the four clades in question is their energy metabolism. The few Apicomplexans studied in this regard rely principally on substrate level phosphorylation at least at some stages of their life cycles (cf., *Plasmodium*, [47]), and some are thought to lack the full complement of the TCA cycle [48]. Chytrids are among the few eukaryotes possessing hydrogenosomes in place of mitochondria [49]–[50], and are almost certainly anaerobes. There is virtually no information on bioenergetics of Labyrinthulids and Jakobids, but both groups are often found in anoxic environments [17], [20], [23]–[24], [30]. This leads us to conclude that the Arctic protistan community studied exhibits an elevated richness of cold-adapted, ancestral protistan lineages with anaerobic/microaerophilic life styles. This is in line with an accepted view that snowball Earth events coincided with global anoxia [5], and the idea that cold environments have been a persistent feature of our planet for at least a part of eukaryotic evolution.

In conclusion, we identified the cold environment of the Arctic as a “hot spot” of protistan biodiversity rich in evolutionarily old lineages. The communities of microbial eukaryotes in temperate habitats appear less diverse and not as rich. Polar environments therefore hold a high potential for future research on microbial discovery. To account for the observed biodiversity patterns, we postulate that cold refuges have persisted throughout the history of eukaryotic evolution, which is in conflict with some current views on the extent of past global warming events.

## Materials and Methods

### Study site and sampling

Sediment samples were collected in July 2003 in Unqussivik, a small bay on Disko Island, Greenland (69°26′03″N; 54°14′13″W). The tidal flat of the bay is composed of gravel and sand of varying porosity. We sampled the fine sand areas (median grain size 1801 µm) with a well developed sulfidic zone below the 5–10 mm depth. Five samples were collected by coring the sediments to 10 cm depth. The samples were transported to the Arctic Station, University of Copenhagen, Qeqertarsuaq, Greenland, and kept at the temperature registered at the time of sampling (6°C) for 12 hr. After thoroughly mixing the samples, we obtained three 3-g subsamples as sources of DNA for PCR reaction employing 3 different primer sets, as described below.

### Nucleic acid isolation and PCR-amplification of 18S rDNA

High molecular weight DNA was obtained from 3-g aliquots of sediment samples as described previously [20]. The sample was heated to 65°C for 2 h in a high-salt extraction buffer (100 mM Tris-HCl [pH8], 100 mM Na_2_EDTA [pH8], 100 mM NaPO_4_ [pH8]) with SDS (20%), CTAB (1%), and Proteinase K (0.1 mg ml^−1^ final). The lysates were purified twice by extraction with an equal volume of chloroform-isoamyl alcohol (24∶1) and precipitated with a 0.6 volume isopropanol. Humic acids and other potential inhibitors of downstream applications were removed by DNA purification with the resin-based Wizard DNA clean up system (Promega, Madison, WI). The integrity of the total DNA was checked by agarose gel electrophoresis (0.8%), and the DNA yield was quantified using a Nanodrop ND-1000 UV-Vis spectrophotometer (Nanodrop Technologies, Wilmington, DE). The molecular weight of extracted genomic DNA was *c.* 13 Kb (as determined by comparison to a molecular weight standard). We extracted 17 µg DNA per g of sediment. Following the multiple-primer approach [23], we amplified fragments of the 18S rRNA gene ranging from ≈1,500 bp to≈1,100 bp, using five different primer sets (Table 2). PCR reactions were performed as described in detail previously [24].

### Clone library construction

The PCR products were used to construct a clone library for each primer set with the pGEM-T Vector System cloning kit (Promega, Madison, WI). According to the primer set identifiers (Table 2), the five clone libraries were designated D1–D5. Plasmids were isolated from overnight cultures using the 96well Directprep kit (Qiagen, Valencia, CA). Nearly 1000 clones evenly distributed among the different primer sets (Table 2), were partially sequenced (M13F sequencing primer) at MWG-biotech (Ebersheim, Germany) using an Applied Biosystems (ABI) 3730 DNA Stretch Sequencer, with the XL Upgrade and the ABI Prism BigDye Terminator version 3.1 Cycle Sequencing Ready Reaction Kit. Initially, partial sequences of each individual primer set were grouped separately into operational taxonomic units (OTUs) based on a 99.0% sequence similarity cutoff value (separately for 5′-3′ and 3′-5′ vector-inserted gene fragments). Selected representative clones of each OTU were then sequenced bidirectionally. Sequence quality assessments, PHRED and PHRAP analysis and assembling were performed with the program CodonCode Aligner v. 1.2.4 (CodonCode corporation, Dedham, MA, USA).

### Sequence clustering

The 18S rDNA sequences were grouped into OTUs based on 99, 98, and 97% sequence similarity cutoff values. This was achieved, as described previously [22], [25], by first making all possible pairwise sequence alignments using ClusatlW at default settings [51] and calculating percent sequence similarities, followed by clustering of the sequences into OTUs using the mean UPGMA linkages as implemented in the OC clustering program (http://www.compbio.dundee.ac.uk/Software/OC/oc.html). The OTU grouping was checked manually to verify that all OTUs were assembled at the cutoff level desired. Potentially chimeric sequences were identified using secondary structure predictions, the Chimera_Check command version 2.7 provided by the Ribosomal database project [52], and partial treeing analyses [53]. Representative gene sequences of each OTU have been deposited in the GenBank database (accession numbers EF100195-EF100415).

### Statistical analyses

Our estimates of species richness are based on sample frequency count data; for mathematical and computational details and recent applications see [22], [25], [54]–[55]. In brief, we assume that each species independently contributes representatives to the sample according to a Poisson process, and that the rates of these processes are randomly generated by a stochastic “abundance” distribution. We currently test seven candidate abundance distributions: point mass or unmixed Poisson (equal species sizes); gamma (negative binomial); inverse Gaussian; lognormal; Pareto, and mixtures of two and of three exponential distributions, fitting these by maximum likelihood using custom software (available on request). Datasets of this type typically have long right tails; we therefore fit every model to every right-truncated subset of each dataset. For each subset we compare models using the Akaike Information Criterion (AIC); across subsets we compare models using an asymptotically-corrected Pearson chi-square goodness-of-fit statistic. We also compute the nonparametric Abundance-based Coverage Estimator [21] and its variants in each case, using the software SPADE [56]. This computationally intensive process is carried out on a supercomputer in the Cornell Theory Center; the work described here entailed about 4,000 separate analyses.

### Phylogenetic analyses

Low quality sequence reads and non-target metazoan sequences were excluded from the phylogenetic analyses. Environmental 18S rRNA gene sequences initially were compared to those in GenBank using gapped BLAST analysis [57] to determine their approximate phylogenetic affiliation. Environmental sequence data together with their closest GenBank matches were compiled in ARB [58] and aligned using the ARB FastAligner utility. Alignments were manually refined using phylogenetically conserved secondary structures. The conserved and unambiguously aligned positions (their number is indicated in Figures 2–10) were used in subsequent phylogenetic analyses. Classification of unique phylotypes was performed via two phylogenetic inference methods: minimum evolutionary distance (ME, Figures 2–10) and maximum parsimony (MP, Figure 1). Trees were constructed using the PAUP* software package 4.0b10 [59]. All heuristic searches were performed using random, stepwise addition of taxa with the TBR branch-swapping algorithm. We applied the program Modeltest [60] to choose the models of DNA substitution that best fit our datasets from among 56 possible models. Modeltest was run for each individual dataset. The DNA substitution models as well as the parameter settings for each tree constructed are described in detail in the legend of the respective figure. We assessed the relative stability of tree topologies using 1000 distance bootstrap replicates. Heuristic searches for bootstrap analyses employed stepwise addition starting trees with simple addition of sequences and TBR branch-swapping.

All datasets used for phylogenetic analyses are available from the authors upon request.
